# Evidence for a large Paleozoic Impact Crater Strewn Field in the Rocky Mountains

**DOI:** 10.1038/s41598-018-31655-4

**Published:** 2018-09-05

**Authors:** Thomas Kenkmann, Kent A. Sundell, Douglas Cook

**Affiliations:** 1 Institut für Geo- und Umweltnaturwissenschaften, Albert-Ludwigs-Universität Freiburg, Baden-Württemberg, Germany; 2 Casper College, Casper, WY USA; 3 Independent Consultant, Colorado Springs, CO USA

## Abstract

The Earth is constantly bombarded by meteoroids of various sizes. During hypervelocity collisions a large amount of energy is coupled to the Earth’s atmosphere leading to disruption of decimeter to hundred meter-sized meteoroids. Smaller meteoroids may form meteorite strewn fields while larger initial bodies and high-strength iron meteoroids may form impact crater strewn fields. Impact crater strewn fields are ephemeral and none documented to date are older than about 63,500 years. Here we report on a newly discovered impact crater strewn field, about 280 Myr old, in tilted strata of the Rocky Mountains near Douglas, Wyoming. It is the oldest and among the largest of impact crater strewn fields discovered to date, extending for a minimum of 7.5 km along a SE-NW trajectory. The apparent width of the strewn field is 1.5 km, but the full extent of the crater strewn field is not yet constrained owing to restricted exposure. We probably see only a small section of the entire crater strewn field. The cascade of impacts occurred in an environment that preserved the craters from destruction. Shock lithification aided this process.

## Introduction

Meteoroids ranging in size from a few tens of centimeters to several tens of meters impact Earth much more frequently than larger meteoroids^1^. The Earth´s atmosphere protects us from the majority of hypervelocity collisions with such small bodies^2^. Once the aerodynamic stresses exceed the strength of a meteoroid it will disrupt while passing through the atmosphere^3,4^. Decimeter to meter-sized meteoroids mostly disintegrate into a number of small fragments that decelerate to terminal velocity and form a meteorite strewn field^5^. The Carancas impact event is an exception where a meter-sized chondritic meteoroid survived atmospheric entry and created a 14 m diameter crater^6^. Decameter-sized objects disrupt violently at high altitudes and can create airbursts that damage infrastructures, but then slow down to terminal velocity when passing through the lower atmosphere. An example is the recent Chelyabinsk event^7^ that formed a 60 km long and less than a kilometer wide meteorite strewn field. Perhaps the largest meteorite strewn field, Gibeon, Namibia, was formed by an iron octahedrite that dispersed over an elliptical area 275 kilometers long and 100 kilometers wide^5^. Atmospheric protection of Earth becomes less effective for more massive meteoroids, and especially for high strength iron meteoroids. Bland and Artemieva^1^ calculated that over the mass range of 10^3^–10^7^ kg, iron impactors transfer ∼3 orders of magnitude more energy to the Earth´s surface than stony meteoroids. While disruption still takes place, a significant portion of their cosmic velocity remains to create impact crater strewn fields^8^. The currently known impact crater strewn fields are Sikhote-Alin^9^, Wabar^10^, Henbury^11^, Kaali^12^, Morasko^13^, and Odessa^14^. A comparison of them is given in Table 1. The lateral spreading in these strewn fields is strongly reduced with respect to meteorite strewn fields and does not exceed one kilometer perpendicular to the trajectory^4^. The craters and crater pits range in size between 10 and 150 m, cluster and overlap, and the biggest is usually situated toward the downrange end of the strewn field ellipse. The term crater pits is referred to those cavities that are formed by strongly decelerated meteoroid fragments that impact at sub-sonic velocity and are not capable of shocking the target. Impact craters formed in quartz rich targets that are larger than 30 m may contain shocked rocks with planar deformation features (PDFs), planar fractures (PFs), and, if the target is porous, impact melt and high pressure polymorphs^15^. Examples are Wabar craters A and B with diameters of 64 and 116 m respectively^16^ and the Gebel Kamil crater with a diameter of 45 m. The hypervelocity impact at Gebel Kamil generated shock characteristics consistent with shock pressures from 30 to 60 GPa^17^. The proper documentation of a shock metamorphic overprint in minerals such as quartz is key for the proof of impact craters^18,19^ and this paper carefully presents such shock features. Meteorites are frequently found in the area of the crater strewn fields (Table 1). All impact crater strewn fields described to date are formed by iron meteoroids and are less than about 63,500 years old^14^ (Table 1). This apparent age limitation is related to the relatively short period of time it takes to either erode or bury such small-scale craters^20^. In some large crater-forming impact events, such as the Meteor Crater (Barringer Crater) event, atmosphere disrupted fragments may impact close to each other and contribute to the formation of a single impact crater^21^.

**Table 1:** Comparison of Impact Crater Strewn Fields.

Crater Strewn Field	Douglas	Wabar^10,16^	Sikhote-Alin^9^	Henbury^11^	Kaali^12,40^	Morasko^13^	Odessa^14^
Age	280 Myr	~150 y	71 y	~4200 y	~3200 y	~5,000 y	63,500 y
Target Lithology	Quartz sand	Quartz sand	Soil	Graywacky	Dolostone	Glacial till	Limestone
Max. Crater Size	~80 m	116 m	26 m	180 m	107 m	60 m	165 m
Number of Craters	40+	3	122	12	7	8	5
Field Length	7 km+	300 m	1.3 km	1.5 km	1 km	1 km	3 km
Field Width	?	100 m	600 m	700 m	?	300 m	1.5 km
Impactor	unknown	Iron IIIAB	Iron Hex.	Iron IIIAB	Iron IAB	Iron Oct.	Iron IAB
Overturned Flap	Yes	No	No	Yes	No	No	No
PDF Lamellae	Yes	Yes	No	No	No	No	No
Impact Glass	(Yes)	Yes		Yes	No		Yes
Shock-lithification	Yes	Yes	No	No	No	No	No

## Results

**Figure 1:**
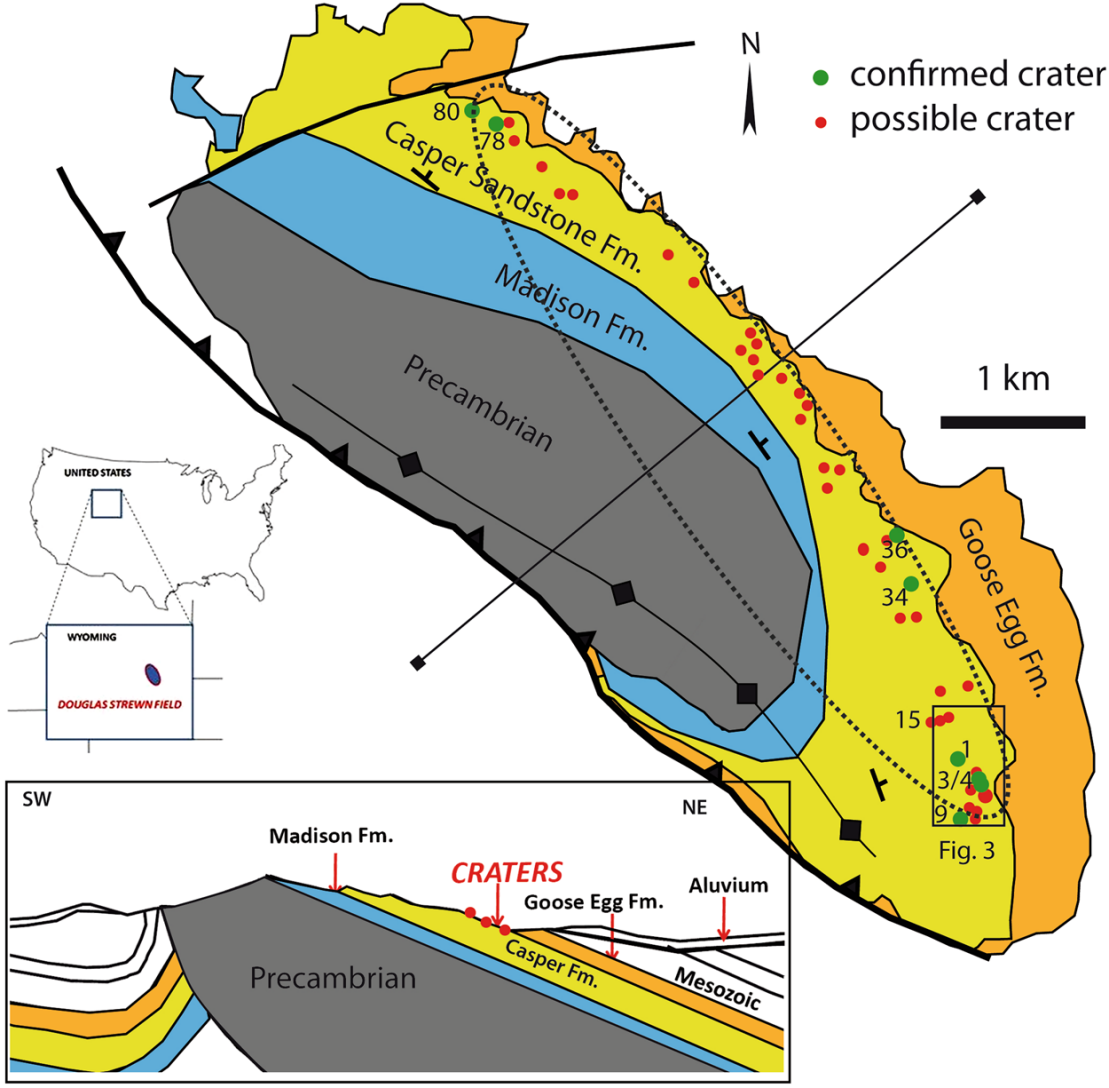
Simplified geological sketch and location of confirmed and possible impact craters at the NE slope of Sheep Mountain anticline, WY, USA. All craters occur in the uppermost Casper Fm. (+/−280 Myr) at the immediate contact to the Goose Egg Formation, Opeche Shale Member. The change in depositional environment may have allowed the shock lithified craters to be submerged and preserved by muds in a quiescent paralic lagoon transgression. The soft mudstone cap was easily eroded recently to expose the shock hardened craters. Note that the entire crater strewn field on the anticline is tilted by about 15°NE (vertical exaggeration of 1.5).

**Figure 2:**
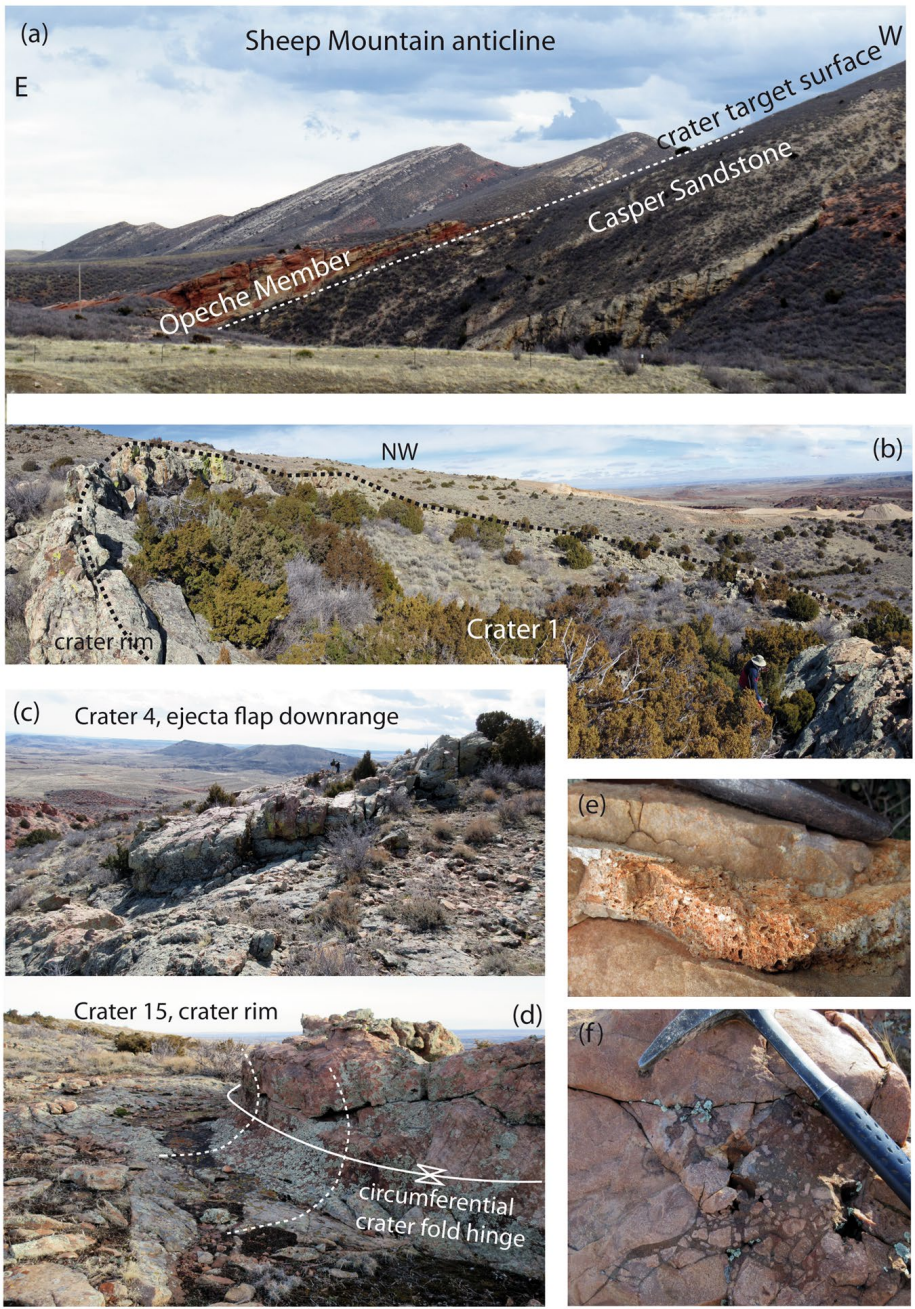
(**a**) Eastern slope of Sheep Mountain anticline. Selective erosion exposed the contact between Casper Sandstone and the Opeche Member of the Goose Egg Formation, where the craters were discovered. (**b**) Panorama view of crater 1, observed from the SSW crater rim. Vantage point is given in Fig. 4a. (**c**) Downrange front of the meter thick, coherent ejecta flap of crater 4. Viewpoint is given in Fig. 4c. (**d**) Partly eroded crater 15 displays uplifted and folded Casper sandstone along the crater rim. (**e**) Injected dike with sub-rounded pebbles and vesicles, (crater 78). (**f**) Pocket with sub-rounded cohesive sand fragments in a fine sand matrix. This type of monomict soft-sediment brecciation occurs frequently along the raised rims of craters 34 and 36.

**Figure 3:**
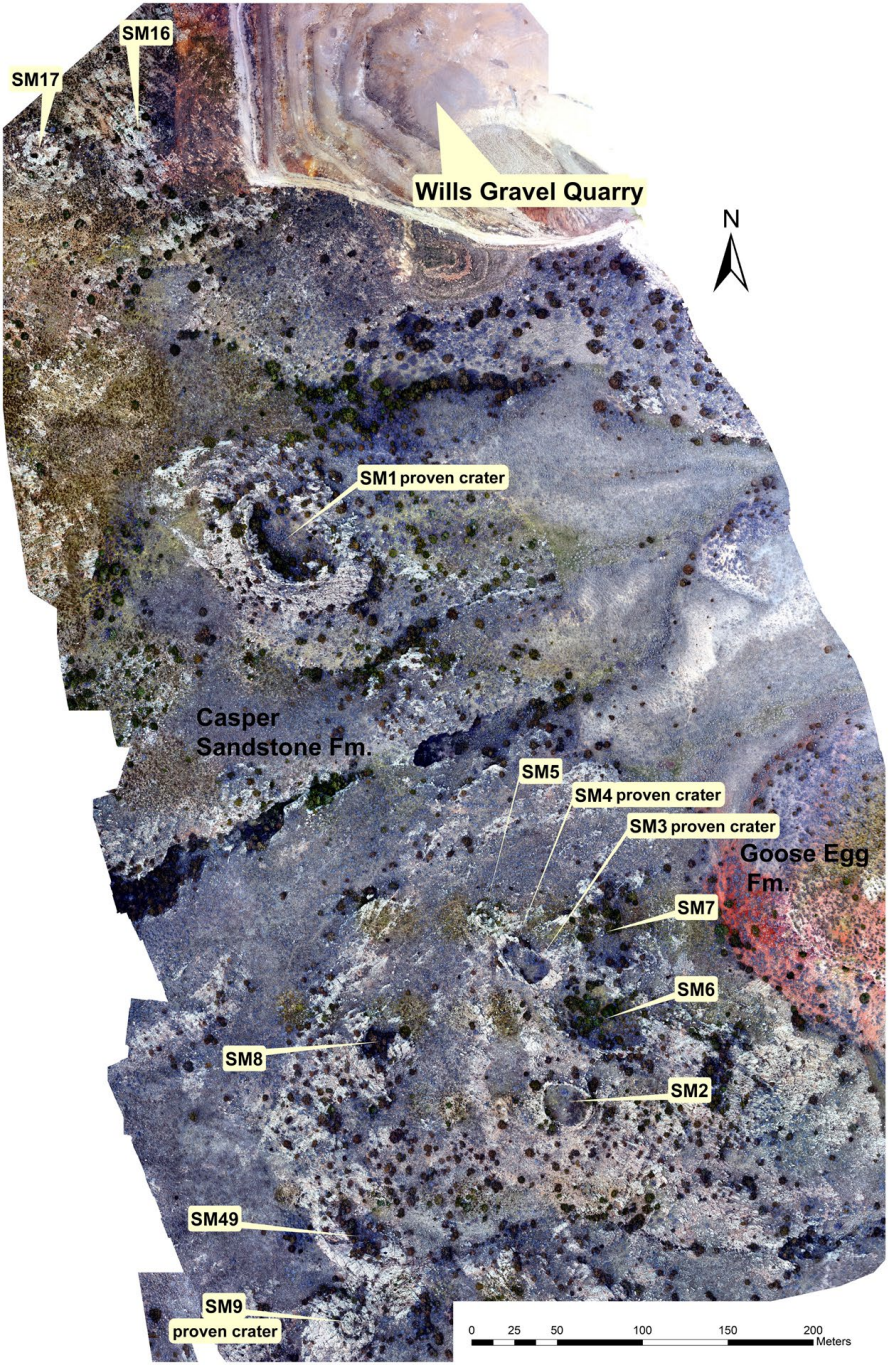
Composite drone imagery of the southeastern crater strewn field. See Fig. 1 for location.

**Figure 4:**
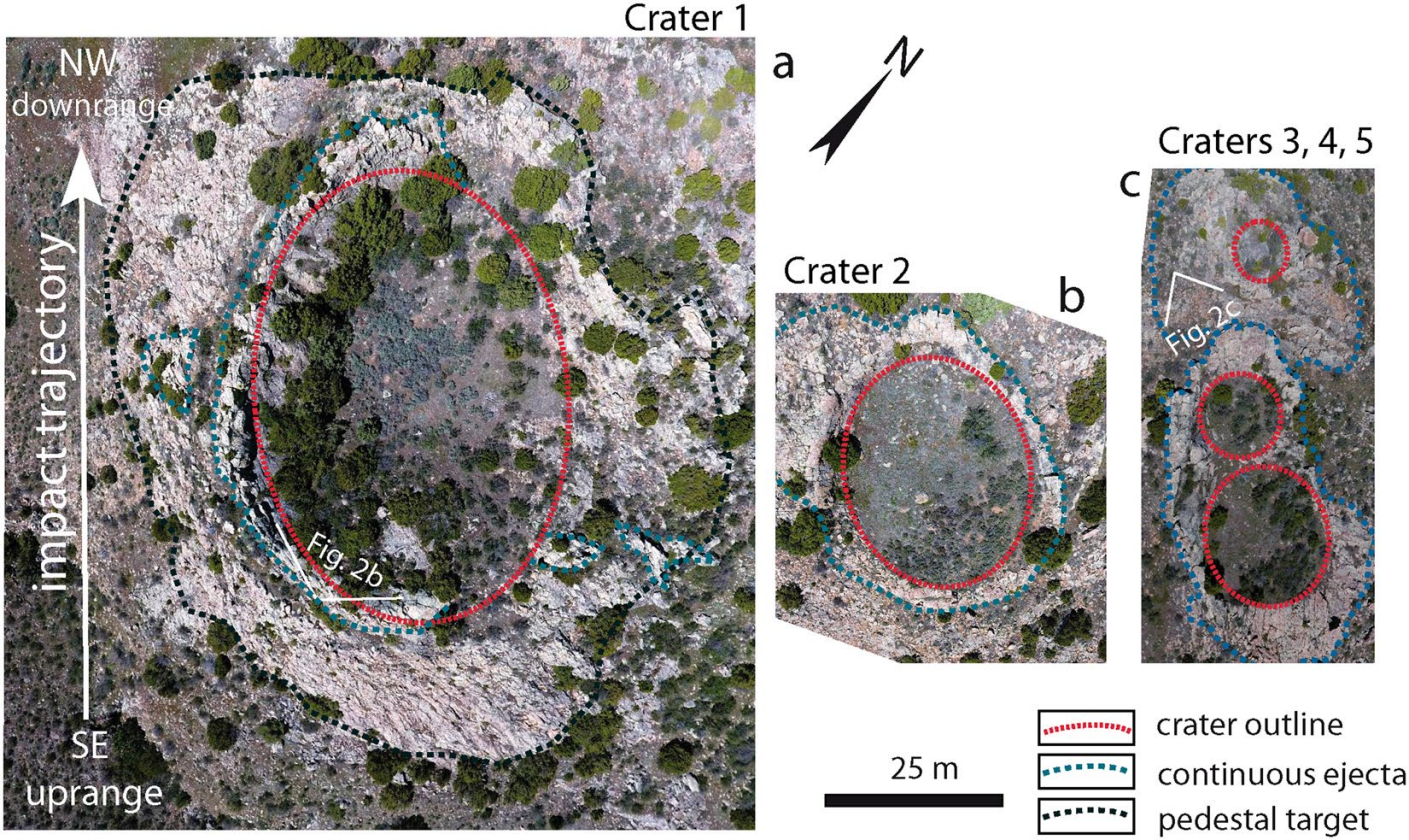
Drone images of craters 1 to 5 (**a**) Crater 1 is 60 m in diameter along the NW-SE trajectory. Its NE flank is eroded. Crater 1 samples have shocked quartz grains. (**b**) Crater 2 has a 31 m long axis and ovoid shape. The apparent overturned flap is well-preserved downrange. (**c**) Craters 3, 4 and 5 interfere with each other and together form a highly elliptical cavity in NW-SE direction. Crater 5 possibly formed by ricochet of the projectile. Dotted red and blue lines outline the crater cavities and the preserved ejecta blankets, respectively.

Here we present a new impact crater strewn field that is exceptional in both size and age. More than 40 circular to ellipsoidal possible impact structures have been identified on the northeast flank of the Sheep Mountain anticline near Douglas, Wyoming, USA (Figs 1, 2a and 3, Table 2) centered on 42°40′38″N, 105°28′00″W. The number of craters given in Table 2 is conservative. Satellite and drone imagery has revealed crater shape, orientation, and size of the craters. The crater structures are exposed stratiform in the uppermost quartz-cemented sandstone of the Permo-Carboniferous Casper Formation. This is a cross-bedded, medium-sorted, low porosity sandstone with an average grain size of 200–300 µm. Owing to pervasive quartz cementation the sandstone almost appears like a quartzite and has a high strength and competence. Except for a few plagioclase grains and local hematite precipitates the sandstone is pure quartzite in composition. Strata were tilted 15° E-NE during the Laramide Orogeny during the Upper Cretaceous and Tertiary (Figs 1 and 2a). Rim-to-rim diameters of the craters and ellipsoidal structures range from 16 meters to ~80 meters (Table 2). Five of these craters were previously reported without conclusive proof of their impact origin^22^. The exposed strewn field on Sheep Mountain has a minimum length of 7.5 km oriented SE-NW (Fig. 1) and includes crater doublets and clusters (Figs 3 and 4). The width of the strewn field is unconstrained. Craters in the SW part of the strewn field are situated at increasing altitude due to tilting of the strata and thus may be eroded from the Sheep Mountain anticline. In contrast, craters in the NE part of the strewn field may be buried beneath younger strata. If we define a strewn field ellipse based on the exposed craters the apparent width is ~1.5 km, defining an ellipticity of five (Fig. 1). Several of the craters that are the least eroded present the geomorphology of a simple impact crater^23^. This includes a raised rim (Figs 2b and 4), an overturned flap, and relics of a continuous proximal ejecta blanket (Figs 2c and 4). Crater 2 has an ovoid shape oriented SE to NW coincident with the apparent strike of the strewn field (Figs 3 and 4). From these features, an impact from SE towards NW can be inferred by comparison to experimentally-formed oblique craters in sand suggesting an impact angle probably even less than 10–15 degrees^24,25^. Craters 3–6 form a chain of craters with SE-NW orientation (Figs 3 and 4c). Crater cavities 3 and 4 overlap each other and form one elongated basin. Crater 5 may be even a result of ricochet of the projectile (Figs 3 and 4c). The only known other terrestrial crater with elliptical outline is Matt Wilson, Australia^26^. The raised rims of the well-preserved craters are a meter or more high and apparently more resistant than the surrounding base level plain (Fig. 2b,d). They are formed by concentrically striking and steeply dipping Casper sandstone. The cross-bedded sandstone shows concave upward foreset beds that merge tangentially with the lower surface and allows distinguishing between normal and overturned attitudes. Several craters expose the fold hinge between the autochthonous crater floor and the overturned flap, among them craters 1, 2, 4, 15 (Fig. 2d), 17, 34, and 36. In structural terms, the overturned flap is the upper limb of an isoclinal recumbent fold with a circumferentially trending fold axis^23^. The most proximal part of the overturned flap forms coherent masses. This is partly visible at craters 1 to 5 (Figs 2c, 3 and 4). However, only relics of the ejecta blankets are preserved and do not allow to infer distribution pattern characteristic for oblique impacts. Due to tilting of the entire target by Laramide tectonics to the NE (Fig. 1), the craters expose a higher western rim (Fig. 2b). Erosion resistance in the crater rim, ejecta blanket, and crater substructure may be caused by shock-lithification^15,16,27^. Several craters show dike injections along the rim and the inner crater slopes (Fig. 2e). Monomict brecciation is well exposed along the raised rims of craters 34 and 36, but the fragments are sub-rounded indicating soft-sediment deformation (Fig. 3f). Some craters are eroded below original base level exposing a pedestal substructure while preserving the original crater (Figs 3 and 4a). Other craters exhibit a lesser quality of preservation and are more deeply eroded. These circular to ellipsoidal structures apparently expose only a remnant substructure and are rated as possible to probable impact craters until shock metamorphism is demonstrated from future field work (Table 2). They may show a diffuse halo with concentric and radial patterns (e.g. Fig. 3, craters SM-16, SM-17).

**Table 2:** Location and size of impact craters of the Douglas crater strewn field.

Crater	Lat.	Long.	Major axis-m	Minor axis-m	NE-SW orientation	Erosion stage	Sampled	Proven	Crater rating
SM-1	42°39′7.30″N	105°26′58.73″W	60	41	x	II	x	x	1
SM-2	42°38′56.45″N	105°26′51.56″W	31	24	x	I	x		1
SM-3	42°38′59.02″N	105°26′52.57″W	17	17	x	I	x	x	1
SM-4	42°38′59.29″N	105°26′52.97″W	12	10	x	I	x	x	1
SM-5	42°38′59.99″N	105°26′53.55″W	10	7	x	I	x		1
SM-6	42°38′58.26″N	105°26′50.80″W	57	40	x	I	x		1
SM-7	42°38′57.06″N	105°26′49.14″W	71			IV			2
SM-8	42°38′57.10″N	105°26′53.01″W	22			IV	x		2
SM-9	42°38′52.29″N	105°26′57.03″W	20			II	x	x	1
SM-11	42°38′41.51″N	105°26′52.13″W	45	36	x	IV	x		2
SM-15	42°39′14.77″N	105°27′5.12″W	26			IV	x		2
SM-16	42°39′14.34″N	105°27′2.87″W	28			IV			2
SM-17	42°39′15.09″N	105°27′2.58″W	27	19	x	IV	x		2
SM-19	42°39′24.70″N	105°26′54.82″W	40			IV			1
SM-20	42°39′23.88″N	105°27′3.99″W	31			IV			2
SM-31	42°39′52.81″N	105°27′26.89″W	63			IV			2
SM-34	42°39′47.45″N	105°27′13.67″W	73	51		I	x	x	1
SM-36	42°40′0.37″N	105°27′17.02″W	21			II	x	x	1
SM-44	42°40′19.27″N	105°27′37.85″W	42	27	x	IV	x		2
SM-45	42°40′20.04″N	105°27′35.81″W	18			IV	x		2
SM-46	42°40′22.73″N	105°27′34.97″W	41			II			1
SM-47	42°38′53.36″N	105°26′47.70″W	82	63	x	IV			2
SM-49	42°38′54.26″N	105°26′57.05″W	51	35	x	IV	x		2
SM-54	42°38′50.95″N	105°26′58.93″W	23			IV			2
SM-58	42°40′18.00″N	105°27′36.36″W	22			IV			2
SM-59	42°40′31.01″N	105°27′51.49″W	28	26		III			1
SM-60	42°40′32.44″N	105°27′51.68″W	15			III			2
SM-61	42°40′37.10″N	105°27′48.58″W	24			I			1
SM-62	42°40′34.30″N	105°27′49.53″W	34			IV			2
SM-64	42°40′30.76″N	105°27′55.40″W	24			IV			2
SM-65	42°40′45.68″N	105°28′2.76″W	37	34	x	III			1
SM-66	42°40′50.15″N	105°28′6.15″W	77			IV			2
SM-67	42°40′53.37″N	105°28′10.31″W	38			IV			2
SM-68	42°40′54.02″N	105°28′7.38″W	36			IV			2
SM-69	42°40′52.58″N	105°28′3.93″W	45	36	x	IV			2
SM-70	42°41′3.82″N	105°28′26.20″W	43			III			1
SM-72	42°41′25.58″N	105°29′8.11″W	42			I			1
SM-73	42°41′25.58″N	105°29′11.78″W	25			I			1
SM-74	42°41′38.84″N	105°29′27.63″W	29			III			1
SM-76	42°41′43.71″N	105°29′35.14″W	41			IV	x		1
SM-77	42°41′42.67″N	105°29′33.81″W	33	22	x	III	x		1
SM-78	42°41′43.71″N	105°29′35.14″W	14			IV	x	x	1
SM-80	42°42′8.98″N	105°29′56.98″W	62	56	x	I	x	x	1

Erosion stage I: crater cavity, overturned flap, ejecta blanket partly preserved, radial and concentric fractures.

Erosion stage II: crater cavity, overturned flap, pedestal morphology due to shock lithification, radial and concentric fractures.

Erosion stage III: crater rim substantially eroded, pedestal morphology partly preserved due to shock lithification.

Erosion stage IV: no crater cavity, possible exposure of crater subsurface, concentric appearance.

RATING: 1 = Compelling 2 = Probable; LOCATION: SM = Sheep Mountain.

**Figure 5:**
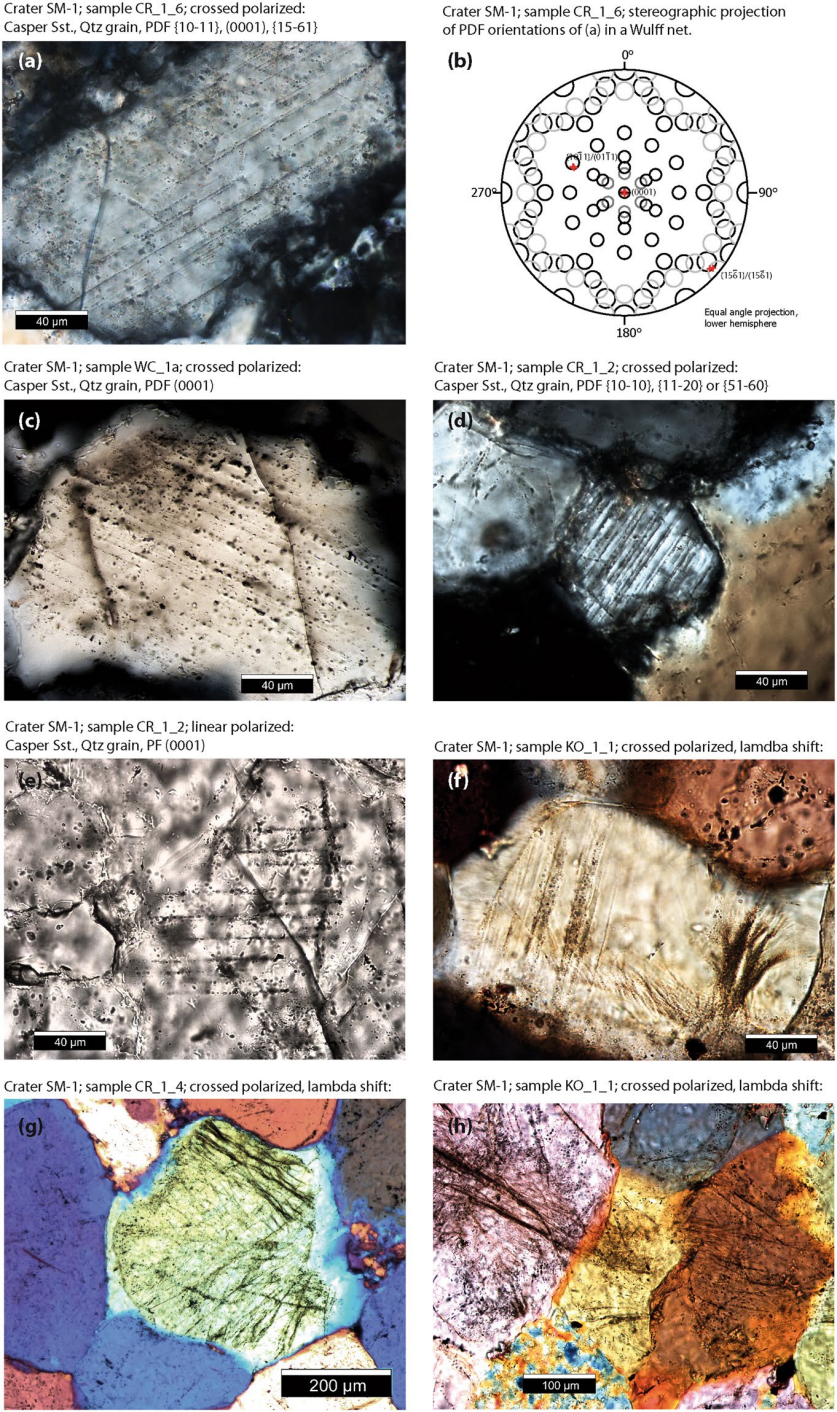
Deformation microstructures in quartz grains of Crater 1. Except for (e) all photomicrographs were taken under crossed polarizers, path difference is added in f, g, and h. (**a**) Cross-cutting and fluid-decorated planar deformation features (PDFs) in quartz grain. Spacing is 3.5 µm on average. (**b**) Crystallographic orientation of lamellae of (**a**). (**c**) Quartz grains with fluid-decorated basal PDF lamellae along (0001), spacing is 5–6 µm. (**d**) Narrow-spaced PDF lamellae parallel to the c-axis. The spacing is 2.5 µm. (**e**) Planar fracture along (0001). (**f**) Concussion fracture (right) is massively decorated with fluid inclusion. Boehm lamellae on the left. (**g**) Grain with high density of fluid-decorated fractures. Note that the fractures end at the round shaped original grain surface and do not extend into the syntactic overgrowth seams suggesting that the impact occurred prior to diagenesis. (**h**) Indentation and interlocking of quartz grains led to shock lithification. Hertzian-type concussion fractures follow point-to-point contacts and stress chains through the grains. These fractures are tensile fractures and are filled with fluid inclusions.

**Figure 6:**
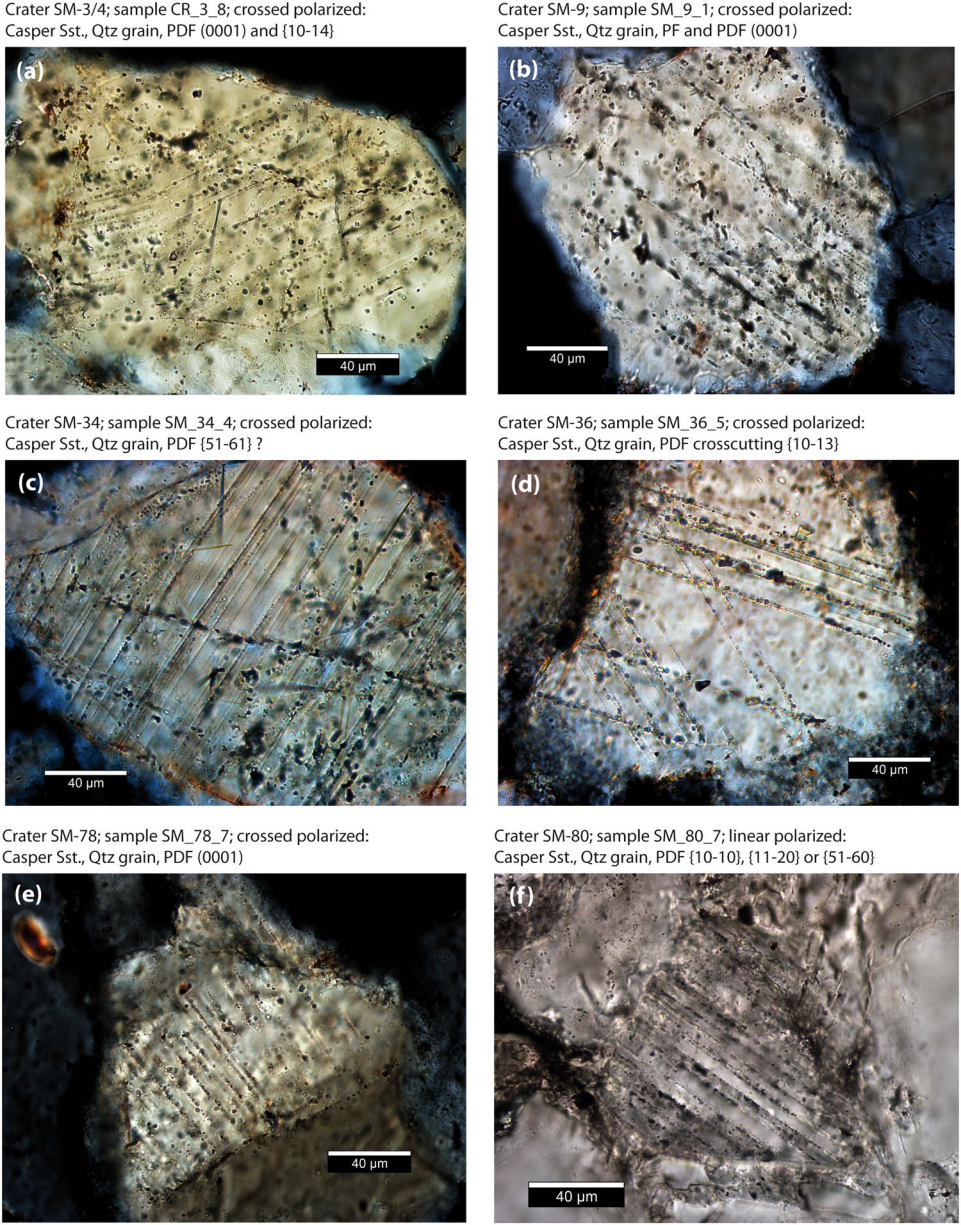
Shock features found in samples of (**a**) doublet crater 3 and 4, (**b**) crater 9, (**c**) crater 34, (**d**) crater 36, (**e**) crater 78, and (**f**) crater 80. For exact location see Table 3.

To date, we have accessed and sampled twenty different craters and investigated 49 thin sections (Table 2). We found shock lamellae in quartz grains along the rim and in the ejecta of seven craters from the SE, central, and NW part of the strewn field (Figs 1, 5 and 6; Table 3). These are crater 1, the doublet crater 3–4, 9, 34, 36, 78, and 80 (Fig. 1). While brittle deformation is common in the samples, shock features are generally rare and restricted to single grains. Table 3 gives an overview of samples that contain shocked grains. Except for one sample, all other samples were taken *in situ*. The fist-sized samples were hammered from the quartzitic Casper sandstone. They were taken from different locations with respect to the given crater as detailed in Table 3. Shocked grains were found in the consolidated ejecta flap downrange and crossrange, in the uplifted crater rim as well as in dikes and breccias of the crater rim (Table 3). We found three grains with two or three orientations and cross-cutting PDF lamellae (Figs 5a,b and 6a,d). A sample from the rim of crater 1 (Figs 2a, 3 and 4a) shows one grain with (0001), {10–11}, and {15–61} lamellae with 3.5 µm average spacing between the lamellae (Fig. 5a,b). Another grain from crater 1 exhibits PDF lamellae parallel to the c-axis at either {11–20}, {10-10}, {51–60} with an average spacing of 2.5 µm between the lamellae (Fig. 5d). Note that due to the absence of a second set, the crystallographic indexing allows no unique indexing here. Basal PDFs along (0001) with 5–6 µm spacing were found in samples from the rim of crater 1 (Fig. 5a,c) as well as from the downrange ejecta flap of the crater doublet 3 and 4 (Fig. 6a), from a dike in the center of crater 9 (Fig. 6b), and from ejecta of crater 78 (Fig. 6e) suggesting a shock metamorphic overprint of less than 10 GPa. Crater doublet 3 and 4 (Figs 3 and 4c) additionally shows a {10–14} set (Fig. 6a). Like in crater 1, {15–61} lamellae were measured in a sample from the rim of crater 34 and planes parallel to the c-axes were also observed in a sample from the ejecta of crater 80. Crosscutting {10–13} sets were recorded from a dike found in the rim of crater 36. Planar Fractures (PFs) with a spacing of 8–20 µm were found in craters 1 and 9. All PDF and PF lamellae are decorated with fluid inclusions.

**Table 3:** Location of samples containing shock features.

Crater	Lat.	Long.	Sample name	Lat.	Long.	Position	Lithology	Shock features		Sampling
								PDF	PF	
SM-1	42°39′7.30″N	105°26′58.73″W	CR_1_2	ca. 42°39′06″N	105°26′55″W	outside crater, crossrange	Casper Sst.	{10–10}, {11–20} or {51–60}	(0001)	*in situ*
SM-1	42°39′7.30″N	105°26′58.73″W	CR_1_6	42°39′06.53″N	105°26′57.70″W	crater rim, uprange	Casper Sst.	(0001); {10–11}; {15–61}		*in situ*
SM-1	42°39′7.30″N	105°26′58.73″W	WC_1a	42°39′07.02″N	105°26′59.22″W	inner crater rim, crossrange	Casper Sst.	(0001)		*in situ*
SM-3/4	42°38′59.32″N	105°26′52.65″W	CR_3_8	42°39′00″N	105°26′53.5″W	ejecta flap downrange	Casper Sst.	(0001); {10–14}		*in situ*
SM-9	42°38′52.29″N	105°26′57.03″W	SM_9_1	42°38′52.40″N	105°26′56.97″W	dike, crater center	Casper Sst.	(0001)	(0001)	*in situ*
SM-34	42°39′47.45″N	105°27′13.67″W	SM_34_4	42°39′46.69″N	105°27′14.13″W	breccia, crest	Casper Sst.	{51–61}?		*in situ*
SM-36	42°40′0.37″N	105°27′17.02″W	SM_36_5	42°39′59.73″N	105°27′16.55″W	dike, uprange	Casper Sst.	{10–13}		*in situ*
SM-78	42°41′43.71″N	105°29′35.14″W	SM_78_7	42°41′47.42″N	105°29′32.45″W	ejecta down-crossrange	Casper Sst.	(0001)		floatstone
SM-80	42°42′8.98″N	105°29′56.98″W	SM_80_7	42°42′08.86″N	105°29′59.11″W	ejecta crossrange	Casper Sst.	{10–10}, {11–20} or {51–60}		*in situ*

Quartz grains are intensely fractured (Fig. 5g,h). Hertzian-type cracks abundantly form along stress chains and grain-to-grain contacts (Fig. 5h). Such concussion fractures are also known from the Wabar craters^10,16^ that formed in a loose sand target, from Coconino sandstone at Meteor Crater^15^, and from MEMIN cratering experiments into wet and dry sandstone^28^. Deformation affected rounded quartz grains but not the quartzitic overgrowth seams (Fig. 5g). This implies that the impact occurred in weakly consolidated to unconsolidated sand prior to diagenesis. Like PDFs, all fractures are massively decorated with fluid inclusions suggesting that the pore space was filled with water during the impact. The presence of water made the sand target cohesive. Ballistic experiments in sand imply that water saturation increases the likelihood of creating a coherent overturned crater flap^29^ as is apparent in the geomorphology of several of the Sheep Mountain craters (Figs 2c and 4). Shock loading of the water-saturated sand target caused shock lithification^27^ of the un-cemented sand and further increased the cohesion of the rocks. Evidence for shock-lithification^27^ can be inferred from microstructure. Most quartz grains lost their original roundness upon impact loading, developed angular corners, and indented grain boundaries. Comminuted grains fit tightly against close-pressing neighbors (Fig. 5f,h). Grains partly interpenetrate one another, adjusting their boundaries to accommodate the curvatures of the intruders. Shock lithified aggregates were also observed at Wabar^10,16^ and in experiments with sandy targets^30^. Melt-agglutination as a further mechanism to lithify the rocks could not yet be demonstrated. Shock-lithification may have contributed to preservation of crater raised rim and ejecta blankets. Some of the craters, in particular crater 1, have pedestal morphologies and appear to preserve and shield the Casper sandstone below from erosion. Apart from shock lithification, this seems to be primarily an effect of a more intense diagenetic sealing of the impact-fractured sand by a quartzitic matrix after the impact event. Pedestal craters represent a class of impact craters unique to Mars that form a plateau surrounded by a well-defined outward-facing scarp. Wrobel *et al*.^31^ suggested that pedestal craters result from combined effects of atmospheric blast and thermal pulse, resulting in the generation of an erosion-resistant armored surface. For the Earth, this effect might be more pronounced as the atmosphere is much denser than on Mars.

In addition to shock metamorphism and intense impact-related fracturing of sand particles of the Casper sandstone, a tectonic overprint associated with the Laramide orogeny during the Cretaceous is obvious in Casper sandstone. Tilting of strata and the formation of the vergent Sheep mountain anticline (Figs 1 and 2a) is followed by strike-slip movements that offset the range along E-W striking faults. These faults contain slickensides and slickenfibres. Casper sandstone shows a fracture cleavage that is related to strata tilting. Shear bands also partly show a preferred orientation parallel the strike of the Sheep Mountain range and are likely of tectonic origin. Boehm lamellae observed at the thin section scale in quartz grains are not indicative for impact but also seem to be related to the later tectonic deformation.

At Sheep Mountain, the target lithology is Casper sandstone, a cross-bedded quartz arenitic sandstone deposited in a mixed eolian, fluvial braided stream, to shoreface environment. The Casper Fm. regionally is a porous and permeable aquifer unit. The impermeable, resistant crater structures occur in the uppermost Casper Fm. and are exhumed from beneath the Permo-Triassic Opeche Shale Member red beds of the Goose Egg Formation (Figs 1 and 2a). These strata are interbedded red to ocher mudstones and siltstones with thin limestone and gypsum stringers indicating an arid paralic to lagoon depositional environment. These mudstones were deposited in a quiescent transgression over the Casper sands. Satellite imagery shows that the craters are present only in this erosion exposed, narrow stratigraphic band along strike at the top dip slope of the Casper Formation (Fig. 1).

## Discussion

The Douglas crater strewn field is exposed along the northeastern flank of Sheep Mountain where Casper Fm. dips parallel to the slope (Figs 1 and 2a). The best preserved craters were found close to the contact with the overlying Goose Egg Fm. If we assume a crater strewn field with an elliptical outline based on the current exposure, its size is 7.5 × 1.5 km. However, possible additional craters on the west side of the ellipse would have been eroded away and additional ones on the east side may lie below the Goose Egg Formation. As a lucky circumstance, the exposure situation of the uppermost Casper Fm. matches the inferred impact trajectory from SE to NW. The latter defines the long axis of the strewn field. The proven craters 1, 3–4, and 9 are concentrated in the SE part of the strewn field ellipse, while craters 34 and 36 are situated in the middle part, and craters 78 and 80 define the NW termination of the observed strewn field (Fig. 1). Further probable crater structures have been found on satellite imagery up to 14 km SW and 19 km NW of Sheep Mountain. These also are on the uppermost Casper Formation on analogous structural and erosional settings to Sheep Mountain. The additional probable crater structures would extend the length of the Douglas Strewn Field to 33 km. However, their impact origin has to be confirmed. Additional field work is planned to explore these structures.

The impact age is inferred to be immediately after Casper Formation deposition and before the deposition of the Goose Egg Formation Opeche Member. This sedimentological boundary indicates that the impact event occurred in the Lower Permian in the Leonardian North American Stage at +/−280 Myr that correlates with the Kungurian stage of the International chronostratigraphic chart^32^. There is no crater filling with younger Casper sandstone in the examined craters. Some craters have some remnant Opeche siltstone preserved in their centers. The deposition of muds apparently started immediately after formation of craters. The original craters would have been eroded away in a short time without shock-lithification, a low-energy transgression of lagoon facies, and eventual burial by Opeche muds. Quiescent conditions with a sedimentation rate about 1–2 mm/yr are typical for modern examples of lagoon environments. However, not all craters are well-preserved (Table 2). For those, a short hiatus between the impact and the onset of sedimentation seems plausible.

The Paleozoic age and the size of the crater field are exceptional and make the Douglas crater strewn field the oldest and among the largest impact crater strewn fields known on Earth. However, the majority of possible craters are allocated to the strewn field by morphological, stratigraphic, and structural analogy to the craters with microstructural proof of impact origin. The regional geology context of scale, symmetry, and stratigraphy discount volcanism, halokinesis, fluid escape, or karst collapse as mechanisms for creating these circular crater pit structures^27^.

The comparison of impact crater strewn fields in Table 1 reveals that Douglas has much in common with the Wabar crater field^16^ except for the size of the field. The unconsolidated sandy target material, the sizes of craters, and the presence of similar deformation microstructures in quartz (concussion fractures, grain indentation, PDFs) along with shock lithification are shared features. In a porous sand target we also expect to find impact melt rock as well as coesite and stishovite similar to Wabar^16^, Kamil^17^, or Meteor crater^15^. Future investigations will focus on this subject. The lack of shock indicators in other strewn fields (Table 1) is partly owing to a different target composition. Recent numerical modeling of the Morasko impact crater strewn field using modeling parameters for a dry sand target^13^ revealed a rock volume shocked to >5 GPa of ~1000 m^3^ for a 65 m diameter crater at an impact velocity of 5.57 kms^−1^. This gives a rough measure of the expected shock volume of crater 1 at Douglas. However the shock volume at Douglas is probably larger as the pore space was water-saturated at the time of impact. Interstitial pore water lowers the impedance contrast between quartz grains and pore space. Less shock wave energy is consumed by the closure of pore space and causes reduced shock wave attenuation with respect to a dry target^33^. The elliptical outline of craters 1–4 indicate a shallow impact angle of less than 15° where even ricochet of the projectile could occur. This indicates that the projectiles have not been decelerated much during their atmospheric traverse. So the impact velocity should be higher than in the mentioned modeling approach^13^.

What is lacking at Douglas so far is the documentation of meteorites, most likely iron meteorites, that are associated with all impact crater strewn fields (Table 1). There are local concentrations of hematite in the uppermost Casper sandstone but such concentrations may have formed during diagenesis. The geochemical identification of the projectile thus poses a further challenge. The chance of preservation of meteorites for a period of 280 Myr is low, in particular for iron meteorites expected for a crater strewn field. But the conservation of Ordovician L-chondrites at Kinnekulle, Sweden^34^, documents that it might be possible for stony meteorites under specific conditions.

The potential outliers of the NW-SE stretching strewn field would increase the width of the impact crater strewn field across the trajectory to 16 km and pose an unprecedented crater strewn field dimension that needs to be addressed in future studies. It is fortuitous that the strewn field orientation based on crater orientation matches with the narrow exposure of the uppermost Casper sandstone. Numerous craters may still be buried under the Opeche muds and younger strata east and north of the exposure. A clustering of the largest craters of the impact crater strewn field is expected downrange to the NW but limited exposure may prevent their identification. Interestingly, the buried 7 km diameter Cloud Creek^35^ impact crater lies exactly downrange on the inferred impact trajectory some 120 km NW of the Douglas crater strewn field. However, the age of this buried crater, constrained by well logging, paleontology, and seismic surveying, is 190+/−20 Myr. So a relationship to the Douglas crater strewn field is ruled out if the age of Cloud Creek is correct. The current state of evidence indicates a large 7.5 × 1.5 km impact crater strewn field formed by a violent disruption of a single body of yet unknown size and composition during passage through the atmosphere. In case the strewn field is wider than a few kilometers alternative scenarios should probably be taken into account. Among them are: (i) The craters represent a section of a secondary crater field around a large, yet unknown crater, that should exist several hundreds of kilometers SE of the current exposure. Secondary craters are intensely studied on the Moon and on Mars^36^. Their origin is obvious if the fields of small craters surround large primary craters and exhibit distinctive morphologies such as shallow, irregular shapes and occurrence in chains and clusters, sometimes with distinctive herringbone patterns. Secondary impact velocities can generate stress pulses exceeding elastic limits of rocks and may cause shock. However, secondary crater fields on Earth are as yet unknown and the geophysical evidence of a large buried crater is so far lacking in the suspected region. (ii) Multiple airbursts of a single meteoroid at a very high altitude may form a very wide, unprecedented impact crater strewn field. The fragmentation process would be affected by the atmospheric density at the 280 Myr impact event. It is known that the Earth experienced a major O_2_ pulse during the Late Paleozoic with an O_2_ concentration of 35%^37^. (iii) A paired meteoroid or an asteroid break-up prior to atmospheric entry or an asteroid shower may have been responsible for a wider extent of the crater field. There are recent examples of asteroids with companions. The Ries-Steinheim impact^37,38^ is an example of a terrestrial paired impact. As long as the extent of the impact crater field is not fully constrained, modeling of the event is not meaningful. Additional craters on Sheep Mountain and outlier areas must demonstrate shocked rocks. This ongoing study will also apply geophysical and geochemical means to address these intriguing questions.

## Methods

Our investigation is based on geological field work, microscopic analysis, and remote sensing using drone and satellite imagery. We used a DJI Phantom 4 drone. The map (Fig. 3) is a compilation of four separate drone flights at 122 m height. The 812 separate images were sewn together using Drone to Map software by DJI and ESRI ARCMAP GIS Programs. We also used geo-referenced satellite images provided by the BaseMap function of ArcGIS 10.1 by ESRI © with spatial resolution of ~ 0.3 m and Google Earth Pro© for identification of crater structures and mapping purposes. Polished thin sections of rock samples were prepared and examined with a LEICA/LEITZ DMR polarizing microscope equipped with a digital camera. Shock lamellae were investigated at 500x magnification. Orientations of planar microstructures in quartz grains, including PFs and PDFs were measured with a LEITZ U-stage mounted onto a LEITZ polarizing microscope. Measurements were then indexed using a template for crystallographic orientations in quartz^39^. The accuracy of U-stage measurements is estimated at ± 5°. Figures were prepared with Adobe© Photoshop and Illustrator CS3.
